# Sex Differences in Suicide Incident Characteristics and Circumstances among Older Adults: Surveillance Data from the National Violent Death Reporting System—17 U.S. States, 2007–2009

**DOI:** 10.3390/ijerph8083479

**Published:** 2011-08-23

**Authors:** Debra Karch

**Affiliations:** Division of Violence Prevention, Centers for Disease Control and Prevention, 4770 Buford Highway NE, MS F-64, Atlanta, GA 30341, USA; E-Mail: DKarch@cdc.gov; Tel.: +1-770-488-1307

**Keywords:** suicide by sex, older adult suicide, public health surveillance, National Violent Death Reporting System, suicide data

## Abstract

Each year in the U.S. more than 7,000 adults aged 60 years and older die of suicide and as the population ages, these numbers are expected to increase. While sex is an important predictor of older adult suicide, differences between males and females are often overlooked due to low occurrence, particularly among women. The National Violent Death Reporting System (NVDRS) bridges this gap by providing detailed information on older adult suicide by sex in 17 US states (covering approximately 26% of the U.S. population). NVDRS data for 2007–2009 were used to characterize male (n = 5,004) and female (n = 1,123) suicide decedents aged 60 years and older, including incident characteristics and circumstances precipitating suicide. Stratification of NVDRS data by sex shows significant differences with regard to the presence of antidepressants (19% and 45% respectively), opiates (18%, 37%), and 14 precipitating circumstances concerning mental health, interpersonal problems, life stressors and a history of suicide attempts. No differences were found for alcohol problems, suicide/other death of family or friends, non-criminal legal problems, financial problems, or disclosure of intent to take their own life. The findings of this study demonstrate the value of using comprehensive surveillance data to understand sex-specific suicide circumstances so that opportunities for targeted prevention strategies may be considered.

## 1. Introduction

Each year over 7,000 adults aged 60 years and older die of suicide in the U.S., accounting for one of every five suicide deaths. Approximately 83% of those deaths between 2005 and 2007 were men, with a rate approximately six times that of women (27.0 and 4.5 per 100,000 population, respectively) [1]. As the population continues to age, the number of suicides by older adults is expected to climb as the “baby boomers” reach older adulthood. A literature search covering the thirty-three years up to 1999 found nearly 10,000 articles on suicide, of which 21% specifically addressed the population 65 years of age and older [2]. Only 3% addressed adults 80 years and older. While it is generally agreed upon that sex is one of the most important predictors of older adult suicide [3,4], a large proportion of these studies looking at suicide risk among older adults don’t comprehensively address the differences between males and females [3,5–8]. This dearth of information on suicide risk by sex may be impacted by factors including the low occurrence of suicide among older adults, particularly among women, who accounted for only 1,100 to 1,200 deaths each year between 1999 and 2005, and declining rates of suicide among women from middle age through older adulthood, the opposite pattern seen with males for whom rates increase with age [1].

Research on sex differences in incident characteristics and circumstances of suicide among older adults is hindered not only by low occurrence levels, but also by insufficient data sets and the limitations of study design methods such as the difficulty in conducting prospective studies in relation to low occurrence levels; recall bias, extensive time commitments and the potential to disturb informants associated with retrospective studies such as psychological autopsies (estimated to average between nearly 2 and 7 hours per decedent); lack of national estimates using survey methodologies; and small sample sizes in both uncontrolled and controlled group designs [3,7–14]. A review by Heisel on studies of late life suicide risk factors found 26 studies, only eight of which had more than 100 cases [12]. Recently Harwood reported studies of the largest sample of persons aged 60 years and older with psychological and physical health problems, but without psychiatric disorder (27 cases), and the largest psychological autopsy study of suicide in older people that has been reported worldwide (54 subjects and 54 controls) [7]. These studies suggested that a broad range of risk and resilience factors must be examined when assessing suicide risk and that further research is needed.

Another important consideration is a lack of common surveillance standards and definitions across studies such as the inclusion age for ‘older adult’ or ‘elderly’ decedents. State and federal agencies, social service groups and older adult organizations use varying benchmarks ranging from 50 to 55, 60 or 65 years of age. This inconsistency, as well as other inconsistent definitions [9,15], makes it difficult to compare results from multiple studies. For the purposes of the current study, we selected the U.S. Centers for Disease Control and Prevention, Division of Violence Prevention standard of adults aged 60 years and older.

While numerous studies have used the aforementioned methodologies to compare older adult suicides to other populations such as younger age groups or other cohorts [16–19], suicide attempters [20,21], and control groups for specific life stressors [7] or psychiatric disorders [6], studies focusing on older adults do not systematically stratify by sex [3]. The few smaller studies that do compare women and men in older age groups have noted mixed results. At least one study found no significant differences between the sexes in suicide of adults over 60 years of age on any demographic, mental or physical health problem or life stressor variable tested [17]. Another found only one significant difference (economic problems were more common among men) between males and females out of seven precipitating circumstances [19]. Others found differences among men and women in a variety of demographic, social, or suicide event variables such as weapon type use, recent somatic illness, living situation and complaints of loneliness [4,22,23]. These mixed results illustrate the difficulty in understanding precipitating factors for suicide in older males compared to female suicide decedents.

An alternative method for capturing information on older adult suicide is through surveillance systems. Surveillance is “the ongoing, systematic collection, analysis, interpretation, and dissemination of data on health related events for use in public health action to reduce morbidity and mortality and to improve public health” [24]. Passive surveillance systems collect information on suicides during the completion of other routine tasks such as filing death certificates. Active surveillance systems seek out suicide cases and actively follow-up and collect information from additional sources [25].

The most widely used passive surveillance system for all deaths including completed suicides is the National Center for Health Statistics’ (NCHS) death certificate data captured in the National Vital Statistics System (NVSS) [26]. NVSS data are limited to information collected on national standardized death certificates which provide, in part, demographics, place of death, and manner and cause of death data. These types of epidemiological surveillance data are useful for mortality studies [27] and provide cause of death information based on the standardized International Classification of Disease, Revision 10 (ICD-10) [28], however, there is limited information to suggest prevention efforts [29]. While the NVSS data set addresses the small case count limitation of other studies it does not capture potential risk factor detail obtained from other study methodologies.

Conwell [27] notes that improvements in surveillance systems for late-life suicidal behavior are needed to “develop the foundation on which to evaluate differences in the elderly subgroup, over time, and in different locations.” Heisel [12] notes that surveillance systems are valuable and can be used to generate hypotheses to be tested in controlled studies. The National Violent Death Reporting System (NVDRS) is an active surveillance system that bridges the gap between pure epidemiological data and the need for collection of contextual and precipitating circumstance information for male and female older adult suicide decedents.

## 2. Methods

### 2.1. Data Source: The National Violent Death Reporting System

The NVDRS is state-based and collects data on all suicides, homicides, legal intervention deaths, unintentional firearm deaths and deaths for which the manner is undetermined [30,31]. State health department staff, with law enforcement and coroner/medical examiner support, collect standardized information with identical definitions across all funded states including demographics of victims of violent death, mechanism of injury, precipitating circumstances, location of injury and death, external cause of injury and ICD-10 codes, autopsy and toxicology results, wound locations, mental health and substance abuse history, special population indicators (e.g., homeless persons, veterans, pregnant women, persons in custodial care) and, for deaths involving a suspect perpetrator, their relationship to the victim [30,32,33].

The NVDRS links data from death certificates (DC), coroner and medical examiner (CME), police/law enforcement (PR), toxicology, and supplemental homicide reports into a single data repository. States manage data collection typically through state health departments or a subcontracted entity, such as a medical examiner’s office. Data are coded by an abstracter, trained to extract data from the various types of reports, or data are imported electronically from other systems (*i.e.*, Bureau of Vital Statistics DC files or CME data sets) and reviewed by the abstractor to ensure accuracy of the codes. Each variable has a strict set of coding requirements to ensure consistency across states [33].

The data used for this analysis include calendar years 2007 to 2009 and represent all data received as of May 2011. Seventeen states (Alaska, California, Colorado, Georgia, Kentucky, Maryland, Massachusetts, New Jersey, New Mexico, North Carolina, Oklahoma, Oregon, Rhode Island, South Carolina, Utah, Virginia and Wisconsin) participated in NVDRS from 2007 to 2009, representing 26% of the U.S. population. In 2010 two additional states, Michigan and Ohio, began data collection but did not meet the 2007 to 2009 data year inclusion criteria for this analysis. Due to the limited number of participating states this data is not nationally representative, however it is the goal of NVDRS to expand to all U.S. states and territories in the future.

Suicide decedents were identified by the abstractor assigned manner of death derived from DC, CME and PR records. Decedents were selected if their age was 60 years or older (n = 6,130) and their sex was known (n = 6,127). Of the 6,127 deaths, 5,004 (81.7%) were male and 1,123 (18.3%) were female.

### 2.2. Variables and Analysis

Comparisons were based on sex and include other demographic characteristics of the suicide decedent (*i.e.*, age, race/ethnicity, marital status), mechanism of injury, location of injury and death, toxicology results, history of suicide attempts, disclosure of intent of kill oneself, the presence or absence of a suicide note, and seventeen other possible precipitating circumstances including: mental health diagnoses and treatment, substance abuse problems, interpersonal problems involving an intimate partner or other person, recent deaths in the family or among friends, problems with other interpersonal violence, and other life stressors (*i.e.*, crisis in the past two weeks, physical health, job, legal and/or financial problems). Stressors are coded as positive when the CME or PR investigation indicated the stressor was believed to have contributed to the decision of the elder to take their own life, a process similar to other studies [17,34]. Statistical analyses were conducted with SPSS 18.0. Frequency distributions and Chi-square tests of significance were conducted for demographic variables, descriptive characteristics of the suicide incident and precipitating circumstances.

## 3. Results

The demographic and incident characteristics of older adults completing suicide are stratified by sex and presented in Table 1. Overall, male and female decedents were most often non-Hispanic whites, 60–64 years of age and married with a mean age of 71.7 years for males and 69.5 years for females (p < 0.001). Statistically significant differences between males and females were found in age group, race/ethnicity, marital status, method of injury, place of death and location of injury (all p ≤ 0.001). Male rates are nearly six times that of females (28.3 and 4.9 per 100,000 population) with the highest rates being among males aged 85 years and older (44.8 per 100,000). Male rates generally increase with age, while female rates remain fairly consistent, varying between 3 and 7 per 100,000 population. Rate ratios of men to women increase from 3.6 to 4.3, 6.2, 8.7, 10.0 and 14.5 from the lowest age group (60–64 years) to the oldest (85 years and older). Non-Hispanic white males had the highest rate (31.7 per 100,000 population) of all racial/ethnic groups by sex for which rates could be computed. Unlike males, the proportion of women who were widowed (32%) was nearly equal to those that were married. Over 80% of both male and female suicidal injuries occurred in the home (10 per 100,000) (Table 1).

The majority of suicides were committed with a firearm, with males at more than 11 times the rate of females (22.1 and 1.9 per 100,000). For males the greatest proportions among mechanisms were firearms (78%), followed by hanging/strangulation/suffocation (10%), while females most often utilized poisoning (41%), followed by firearms (38%) (Table 1). Poisoning deaths accounted for only 7% of male suicides and, in addition, the type of poisons most often utilized by males and females differed. Of the poisoning deaths, males most often died from prescription drug overdose (43%) followed by inhaling carbon monoxide or other gases (32%). Females most often died of prescription drug overdose (64%) and over the counter drug overdose (11%) (Table 2).

The results of toxicology testing following suicide are presented in Table 3. Females were tested more often than males for all drug types including alcohol, amphetamines, antidepressants, cocaine, marijuana and opiates. Of those tested, females were more likely than males to be positive for antidepressants (45% and 19%, respectively) and for opiates (37% and 18%, respectively). The percent of positive tests for alcohol, amphetamines, cocaine and marijuana were nearly equal in both sexes.

Precipitating circumstance data presented in Table 4 indicate that there were no significant differences between males and females in the areas of alcohol problems, friend or family suicides or deaths in the past five years, interpersonal violence victimization in the past month, civil legal problems, financial problems or their disclosure of intent to take their own lives.

Nearly half of male decedents (45%) were reported as having a depressed mood by family, friends or other informants compared to 41% of females (p = 0.027). Significantly more females (58%) than males (35%) had a diagnosed mental health problem (p ≤ 0.001) and were in current mental health treatment (81% and 72% respectively, p ≤ 0.001). Approximately 80% of both males and females with a current mental health problem had a diagnosis of depression/dysthymia, with anxiety disorders (males = 9%, females = 12%) and bipolar disorder (males = 6%, females = 11%) occurring in smaller proportions (Table 5). Statistically significant differences were found between males and females for two diagnoses: males having higher proportions of PTSD (p = 0.006) and females having higher proportions of bipolar disorder (p ≤ 0.001).

Less than 10% of both male and female decedents were believed to have an alcohol problem and males were less likely than females to abuse other substances (2% and 5% respectively, p < 0.001). Males were more likely than females to have intimate partner problems (12% and 8% respectively, p ≤ 0.001), while females were more likely to have interpersonal problems with someone other than an intimate partner (9% and 5%, p < 0.001). Males were also more likely than females to have been perpetrators of interpersonal violence in the past month (2.7% and 0.6%, p < 0.001), have experienced a recent crises in the past two weeks (24% and 18%, p < 0.001), and have physical health (54% and 43%, p ≤ 0.001), job (5% and 3%, p < 0.001) or criminal legal problems (4% and 1%, p < 0.001). Males were less likely than females to leave suicide notes (30% and 42% respectively, p ≤ 0.001) and to have a history of suicide attempts (9% and 25%, p < 0.001).

## 4. Discussion

Surveillance-based demographic characteristics for older adult suicides are understandably different from other types of studies, primarily due to small sample sizes and lack of random sampling in the other studies. The NVDRS surveillance system relies on the capturing of all suicides in funded states and thus is not subject to either of those limitations. One important demographic difference in the NVDRS is the percent of male and female decedents. In previous studies male sex ranged from approximately 45% to 95%, [5,8,17,35–37] while NVDRS more closely mirrors, but is still not representative of the entire U.S. population due to the fact its data collection only covers seventeen states. Because so few studies compare male and female differences, and have defined age groups and circumstance variables inconsistently, comparison of their findings with NVDRS is difficult. Despite these differences, there are clear similarities in non-demographic surveillance data and data obtained from other types of studies.

Similar to previous research, this study found that the home was the most common location of injury among older adults [2] and of both sexes [37]. Firearms were the most common mechanism with nearly twice as many males using this method than females [1,3,35,38–40]. Poisoning and hanging/strangulation/asphyxiation were also common [41]. Also consistent with previous research, physical illness was a common precipitator [2,8,17,37,40,42] while financial or legal problems were evident in only a small percentage of cases [17]. Less than one in four older adult suicide decedents have made known prior attempts [9,17,27,43] and disclosure of intent to take their own life was infrequent [2]. Canetto [3] notes that the percent disclosing intent to take their lives may be similar among males and females. Mental health problems, especially depression [40,44,45], were common and alcohol and drug use disorders were noted with less frequency [2,3,5,9]. Females were more likely to seek psychiatric treatment than males [3] and females are more likely to have a history of mental health treatment [5]. Few studies report on actual diagnosis by sex [3]. Death of someone close to the suicide decedent was noted as a contributor in a small percentage of cases [17].

Data that are reported separately for males and females in other studies closely mirror NVDRS data in many ways. For example, Turvey [8] presented data on 20 suicide decedents 60 years of age or older, only one of whom was female. The study does not separate the one female from the males but, with that limitation, found that precipitating circumstance data are consistent with NVDRS male only data; that is, economic/financial problems (7% for each) and legal problems ranging from 2% to 3%. In addition, males and females are similar in disclosure of their intent to end their lives [3], a finding also noted in NVDRS (28% and 25% respectively). Additionally, a separate study reports an average of 1.99 stressors per male and 1.98 for female decedents [19]. NVDRS notes 1.65 for males and 1.56 for females; similar ratios of male to female stressors.

Additional precipitating interpersonal and life stressor circumstance information was more difficult to compare due to varying definitions of similar terms and the inconsistent collapsing of multiple circumstances into broad categories; for instance, in one study financial problems includes not only monetary issues but also job and unemployment problems [17] while in other studies, and NVDRS, those are evaluated separately. Using Carney’s definition, financial problems were noted in 12% of suicides over the age of 60 while in NVDRS, combining the financial and job problem circumstances resulted in 11% [17].

Adding to the literature, the differences in demographic, incident and precipitating circumstance data for older males and females who take their own lives are presented on a population-based level with large sample sizes and consistent definitions across states. Significant differences in proportions of older male and female suicide decedents are noted for age group, race ethnicity, marital status, method of injury, place of injury, place of death, toxicology, and problems with mental health and substance abuse, interpersonal relationships, physical health, jobs, and legal issues. These data were previously unavailable on a large scale.

Comparability of these findings to other studies looking at sex differences is complicated by differing case finding and variable definitions. For instance, NVDRS captures the manner of death (*i.e.*, suicide, undetermined, unintentional firearm, homicide and legal intervention) in a standardized way from both the DC and CME and retains them in separate variables. Manners of death can be analyzed separately or in aggregate without jeopardizing the size of the study. In other research differences were noted among studies in their inclusion of numerous combinations of manner of death in assessing suicide. For some, determination of suicide and thus inclusion in a study was based only on a DC manner of suicide. Other studies include a CME determination of suicide, while still others include suicides, undetermined and accidental deaths that appeared as though they could have been suicides [7]. Standardization of case finding and variable definitions in a large scale study enables the analysis not only of sex differences at a sizeable aggregate level but also in conjunction with other variables such as state or county of occurrence where prevention programming and legislative support can be provided.

Active surveillance methods overcome a number of other limitations of controlled studies and can substantially increase death counts for study, especially with marginalized groups such as older adults who take their own lives. The NVDRS takes advantage of the availability of existing documents and, by consolidating the information into single case records, produces a more complete picture of suicide incidents than would be obtained by single source or limited access systems. Although CME [46] and PR are not standardized at the agency level, the NVDRS methodology of multiple source documents improves our ability to understand the circumstances surrounding older adult suicides.

The differences found in this study between older adult males and females who take their lives may be related to the gender roles and the unique experiences of aging. The National Strategy for Suicide Prevention, Goals and Objectives for Action suggests a number of strategies to reduce suicidal behavior including promoting awareness, reducing the stigma of seeking care for mental health and substance abuse problems, implementing community based programs, reducing access to lethal means, and providing training for practitioners who care for the elderly [47]. Older adult males in particular may benefit greatly from programs that seek to keep them active in later life, facilitate their access to mental health services, assist them in dealing with immediate crises, and evaluate their access to highly lethal weapons. Older adult females on the other hand, may benefit greatly from services to assist them in the earlier stages of the aging process, dealing with widowhood, and close monitoring of prescription medications, particularly opiates. Regardless of sex, an immediate and comprehensive response to suicidal ideation and previous suicide attempts is critical as nearly one in four older adults disclose their intent to take their lives before doing so. The effectiveness of programs designed to mitigate or respond to these issues is key to prevention.

Evaluation of the effectiveness of prevention programs for older adult suicide remains challenging for the methodological reasons presented earlier in this paper. Public health entities employing primary and secondary prevention strategies and utilizing strict scientific methods are ideal partners in bridging this knowledge gap in the future.

Primary prevention strategies that have been explored include efforts such as training professional and lay persons having frequent contact with older adults on risk and protective factors, community resources, and recognition of the warning signs of potential suicidal behavior; promoting physical activity and ensuring affordable medical coverage for physical and mental health needs; reducing isolation among elders by promoting participation in community events; and balancing work and relationship commitments as well as others [3,48]. More targeted than primary prevention efforts, secondary prevention efforts have included the provision of therapeutic interventions for mental health and other life crises, case management services, transportation assistance to reach service providers, and monitoring of prescription drug and substance abuse. First and foremost however, is the need to identify which of the many potential strategies are the most effective for suicide prevention among older adult males and for females to ensure the unique needs of these two groups are met; a challenge for the field of public health.

The results from this study are subject to at least six limitations. First, while we identified precipitating circumstances for both males and females, we were not able to discern the intensity of those circumstances or which circumstances were most contributory. Second, it was not possible to determine if male and female suicide decedents have more life stressors than the other male and female older adults who do not die by suicide. Third, the inability to request additional follow-up detail on specific circumstances and to understand chronological sequencing of circumstances inhibits temporal analysis for both sexes. Fourth, there are other potentially important aspects of suicide circumstances that are not included in the NVDRS. The concepts of social isolation, personality traits and level of functional impairment, for example, have all been suggested as contributory in other suicide studies among the elderly but are often not measures reported in CME or PR [2]. Fifth, toxicology results are limited to decedents who were tested for each substance and are not generalizable to all suicide decedents. Sixth, NVDRS surveillance data represent only 17 U.S. states, comparisons in this paper are limited only to other studies using U.S. data. Results from international studies [40], while just as important, may reflect differing cultural factors, aging experiences, life course events, effects of demography and differences in clinical profiles and divergent motivations that make them fundamentally different than U.S. data.

## 5. Conclusions

This study found significant differences by sex in age group, race ethnicity, marital status, method of injury, place of injury, place of death, toxicology, and problems with mental health and substance abuse, interpersonal relationships, physical health, jobs, and legal issues for adults aged 60 years and older who took their own lives. In addition, it demonstrates the ability to capture a wide variety of demographic, incident and precipitating circumstance data utilizing surveillance methods. NVDRS dramatically increased the number of cases for analysis by sex and thus will also allow for sex-based analysis of specific older adult subpopulations (*i.e.*, Native Americans, inmates, homeless persons). A better understanding of the multi-faceted differences between male and female older adults who take their own lives can help to develop a more comprehensive understanding of specific outcomes so that targeted prevention strategies may be implemented.

## Figures and Tables

**Table 1.** Number, percentage and rate^∞^ of suicides of adults aged 60+ years, by sex, age group, race/ethnicity, marital status, method, location where injury occurred and place of death—National Violent Death Reporting System, 17 states †, 2007–2009.

	**Male**			**Female**			**Total**			**Rate Ratio**
**Characteristic**	**No.**	**%**	**Rate**	**No.**	**%**	**Rate**	**No.**	**%**	**Rate**	**M:F**
**Age group (years)**										
60–64	1,404	28.1	25.5	419	37.3	7.0	1,823	29.8	15.9	3.6
65–69	962	19.2	24.2	253	22.5	5.6	1,215	19.8	14.3	4.3
70–74	772	15.4	25.6	153	13.6	4.1	925	15.1	13.8	6.2
75–79	767	15.3	32.2	121	10.8	3.7	888	14.5	15.8	8.7
80–84	587	11.7	35.9	95	8.5	3.6	682	11.1	15.9	10.0
85+	512	10.2	44.8	82	7.3	3.1	594	19.7	15.5	14.5
**Total**	**5,004**	**100**	**28.3**	**1,123**	**100**	**4.9**	**6,127**	**100**	**15.1**	**5.8**
**Race/ethnicity**										
White, non-Hispanic	4,647	92.9	31.7	1,048	93.3	5.6	5,695	93.0	17.1	5.7
Black, non-Hispanic	167	3.3	9.8	19	1.7	§	186	3.0	4.3	§
Asian/Pacific Islander	57	1.1	14.2	35	3.1	7.1	92	1.5	10.3	2.0
American Indian/Alaskan Native	28	0.6	18.3	10	0.9	§	38	0.6	11.0	§
Hispanic	102	2.0	14.4	11	1.0	§	113	1.9	7.1	§
Other/Unknown	3	0.1	§	0	0.0	§	3	0.0	§	§
**Total**	**5,004**	**100**	**28.3**	**1,123**	**100**	**4.9**	**6,127**	**100**	**15.1**	**5.8**
**Marital Status¶**										
Married	2,537	50.7	NR	393	35.0	NR	2,930	47.8	NR	NR
Never Married	331	6.6	NR	54	4.8	NR	385	6.3	NR	NR
Widowed	951	19.0	NR	354	31.5	NR	1,305	21.3	NR	NR
Divorced	1,054	21.1	NR	284	25.3	NR	1,338	21.8	NR	NR
Married, but Separated	7	0.1	NR	2	0.2	NR	9	0.1	NR	NR
Single, not otherwise specified	21	0.4	NR	8	0.7	NR	29	0.5	NR	NR
Unknown	103	2.1	NR	28	2.5	NR	131	2.1	NR	NR
**Total**	**5,004**	**100**	NR	**1,123**	**100**	NR	**6,127**	**100**	NR	**NR**
**Method**										
Firearm	3,907	78.1	22.1	424	37.8	1.9	4,331	70.7	10.7	11.6
Sharp instrument	90	1.8	0.5	29	2.6	0.4	119	1.9	0.3	1.3
Blunt instrument	7	0.1	§	0	0.0	§	7	0.1	§	§
Poisoning	368	7.4	2.1	462	41.1	2.0	830	13.5	2.1	1.1
Hanging, Strangulation, Suffocation	500	10.0	2.8	145	12.9	0.6	645	10.5	1.6	4.7
Explosive	1	0.0	§	0	0.0	§	1	0.0	§	§
Fall	44	0.9	0.2	25	2.2	0.1	69	1.1	0.2	2.0
Drowning	44	0.9	0.2	24	2.1	0.1	68	1.1	0.2	2.0
Fire/Burns	3	0.1	§	5	0.4	§	8	0.1	§	§
Motor vehicle including buses, motorcycles and other transport vehicles	21	0.5	0.1	7	0.6	§	28	0.4	0.1	§
Other	13	0.3	§	1	0.1	§	14	0.3	§	§
Unknown	6	0.1	§	1	0.1	§	7	0.1	§	§
**Total**	**5,004**	**100**	**28.3**	**1,123**	**100**	**4.9**	**6,127**	**100**	**15.1**	**5.8**
**Location**										
House	4,175	83.4	23.6	951	84.7	4.2	5,126	83.7	12.7	5.6
Street/road, sidewalk, alley	115	2.3	0.7	16	1.4	§	131	2.1	0.3	§
Motor vehicle	84	1.7	0.5	18	1.6	§	102	1.7	0.3	§
Commercial/Retail Area	27	0.6	0.2	2	0.2	§	29	0.4	0.1	§
Industrial or construction area	17	0.3	§	0	0.0	§	17	0.3	§	§
Office building	15	0.3	§	1	0.1	§	16	0.3	§	§
Parking lot/public garage	53	1.1	0.3	6	0.5	§	59	1.0	0.1	§
Abandoned house/warehouse	5	0.1	§	0	0.0	§	5	0.1	§	§
Sports/athletic area	68	1.4	0.4	6	0.5	§	74	1.2	0.2	§
Hospital or medical facility	24	0.5	0.1	14	1.2	§	38	0.6	0.1	§
Supervised residential facility	16	0.3	§	8	0.7	§	24	0.4	0.1	§
**Location**										
Farm	27	0.5	0.2	4	0.4	§	31	0.5	0.1	§
Jail/prison/detention facility	10	0.2	§	0	0.0	§	10	0.2	§	§
Natural area (field, river, beach, woods)	143	2.9	0.8	24	2.1	0.1	167	2.7	0.4	8.0
Hotel/Motel	47	0.9	0.3	16	1.4	§	63	1.0	0.2	§
Railroad tracks	8	0.2	§	3	0.3	§	11	0.2	§	§
Other	94	1.8	0.5	15	1.3	§	109	1.7	0.3	7.2
Unknown	76	1.5	§	39	3.5	§	115	1.9	0.3	§
**Total**	**5,004**	**100**	**28.3**	**1,123**	**100**	**4.9**	**6,127**	**100**	**15.1**	**5.8**
**Place of Death**										
Hospital inpatient	301	6.0	1.7	121	10.8	0.5	422	6.9	1.0	3.4
ED/outpatient	266	5.3	1.5	60	5.3	0.2	326	5.3	0.8	7.5
Dead on arrival	86	1.7	0.5	15	1.3	§	101	1.6	0.2	§
Hospice facility	12	0.2	§	1	0.1	§	13	0.2	§	§
Nursing home/long term care facility	45	0.9	0.3	7	0.6	§	52	0.8	0.1	§
Decedent’s home	3,472	69.4	19.7	769	68.5	2.1	4,241	69.2	10.5	9.4
Other injury site (not decedent’s home)	744	14.9	4.2	138	12.3	0.5	882	14.4	2.2	8.4
Unknown	78	1.6	§	12	1.1	§	90	105	§	§
**Total**	**5,004**	**100**	**28.3**	**1,123**	**100**	**4.9**	**6,127**	**100**	**15.1**	**5.8**

∞ Per 100,000 population.

† Alaska, Colorado, Georgia, Kentucky, Maryland, Massachusetts, North Carolina, New Jersey, New Mexico, Oklahoma, Oregon, Rhode Island, South Carolina, Utah, Virginia, Wisconsin and four California counties.

§ Rates not reported when number of decedents is < 20.

Includes only decedents aged ≥ 18 years.

NR Not Reported. Rates cannot be computed for marital status because denominators are unknown.

**Table 2.** Number and percentage of suicides by poisoning by type of poison—National Violent Death Reporting System, 17 states, ^∞^ 2007–2009.

	**Male**		**Female**		**Total**	
**Type of Poison**	**No.**	**%**	**No.**	**%**	**No.**	**%**
Alcohol only	10	2.7	10	2.2	20	2.4
Prescription drugs only	159	43.2	297	64.3	456	54.9
Over the counter drugs only	25	6.8	49	10.6	74	8.9
Carbon monoxide/other gas only	118	32.1	35	7.6	153	18.4
Alcohol and prescription drugs	9	2.4	9	1.9	18	2.2
Prescription and over the counter drugs	8	2.2	18	3.9	26	3.1
Other specified substance	15	4.1	11	2.4	26	3.1
Other combination of substances	3	0.8	3	0.6	8	0.9
Unknown	21	5.7	30	6.5	51	6.1
**Total**	**368**	**100**	**462**	**100**	**830**	**100**

∞ Alaska, Colorado, Georgia, Kentucky, Maryland, Massachusetts, North Carolina, New Jersey, New Mexico, Oklahoma, Oregon, Rhode Island, South Carolina, Utah, Virginia, Wisconsin and four California counties.

**Table 3.** Number and percentage of suicides of adults aged 60+ years tested for alcohol and drugs whose results were positive—National Violent Death Reporting System, 16 states, ^∞^ 2007–2009.

	**Male**				**Female**				**Total**				***p*-value**
	**Tested**		**Positive**		**Tested**		**Positive**		**Tested**		**Positive**		
**Toxicology variable**	**No.**	**%**	**No.**	**%**	**No.**	**%**	**No.**	**%**	**No.**	**%**	**No.**	**%**	
Blood Alcohol Concentration (BAC)†	3,012	60.2	602	20.0	743	66.2	139	18.7	3,755	61.3	741	19.7	0.755
Alcohol ≤ 0.08 g/dL†			213	35.4			62	44.6			275	37.1	-
Alcohol > 0.08 g/dL†			353	58.6			69	49.6			422	57.0	-
Alcohol Positive–Level Unknown			36	6.0			8	5.8			44	5.9	-
Amphetamines	1,659	33.2	21	1.3	503	44.8	3	0.6	2,162	35.3	24	1.1	0.401
Antidepressants	1,532	30.6	284	18.5	486	43.3	219	45.1	2,018	32.9	503	24.9	< 0.001
Cocaine	1,675	33.5	19	1.1	538	47.9	2	0.4	2,213	36.1	21	0.9	0.351
Marijuana	1,336	26.7	13	1.0	382	34.0	1	0.3	1,718	28.0	14	0.8	0.502
Opiates	1,691	33.8	296	17.5	560	49.9	207	37.0	2,251	36.7	503	22.3	< 0.001

∞ Alaska, Colorado, Georgia, Kentucky, Maryland, Massachusetts, North Carolina, New Jersey, New Mexico, Oklahoma, Oregon, Rhode Island, South Carolina, Utah, Virginia, Wisconsin.

† The alcohol variable reflects the blood alcohol content (BAC) of victims using 0.08% as the standard for intoxication. The other substances are indicated if there are any positive results. The levels for these substances are not measured.

**Table 4.** Number ^∞^ and percentage † of suicides of adults aged 60+ years, by associated circumstances and sex—National Violent Death Reporting System, 17 states, § 2007–2009.

	**Male**		**Female**		**Total**		***p*-value**
**Associated circumstances**	**No.**	**%**	**No.**	**%**	**No.**	**%**	
**Mental Health/Substance Abuse**							
Current Depressed Mood	1,879	44.5	392	40.6	2,271	43.8	0.027
Current Mental Health Problem	1,490	35.3	561	58.1	2,051	39.5	< 0.001
Current Mental Health Treatment¶	1,077	72.3	454	80.9	1,531	74.6	< 0.001
Ever Treated for Mental Health Problem	1,276	30.2	511	52.9	1,787	34.4	< 0.001
Alcohol Problem	415	9.8	80	8.3	495	9.5	0.140
Other Substance Abuse Problem	100	2.4	48	5.0	148	2.9	< 0.001
**Interpersonal**							
Intimate Partner Problem	508	12.0	79	8.2	587	11.3	< 0.001
Other Relationship Problem (non-intimate)	230	5.4	89	9.2	319	6.1	< 0.001
Suicide of Family Member or Friend within Past Five Years	44	1.0	9	0.9	53	1.0	0.758
Other death of Family Member or Friend within Past Five Years	399	9.5	92	9.5	491	9.5	0.944
Perpetrator of Interpersonal Violence within Past Month	116	2.7	6	0.6	122	2.4	< 0.001
Victim of Interpersonal Violence within Past Month	4	0.1	1	0.1	5	0.1	0.937
**Life Stressor**							
Crisis in Past Two Weeks	991	23.5	169	17.5	1,160	22.4	< 0.001
Physical Health Problem	2,267	53.7	417	43.2	2,684	51.7	< 0.001
Job Problem	216	5.1	24	2.5	240	406	< 0.001
Recent Criminal Legal Problem	172	4.1	13	1.3	185	3.6	< 0.001
Non-criminal Legal Problem	63	1.5	10	1.0	73	1.4	0.277
Financial Problem	398	9.4	83	8.6	481	9.3	0.420
**Suicide Event**							
Left a Suicide Note	1,352	30.2	407	42.1	1,759	33.9	< 0.001
Disclosed Intent to Commit Suicide	1,186	28.1	250	25.9	1,436	27.7	0.166
History of Suicide Attempt(s)	358	8.5	237	24.5	595	11.5	< 0.001

∞ N = 5,188 (4,222 males and 966 females). Circumstances were not known for 939 deaths.

† Percentages might exceed 100% because multiple circumstances might have been coded.

§ Alaska, Colorado, Georgia, Kentucky, Maryland, Massachusetts, North Carolina, New Jersey, New Mexico, Oklahoma, Oregon, Rhode Island, South Carolina, Utah, Virginia, Wisconsin and four California counties.

Includes only those with a current mental health problem.

**Table 5.** Number^∞^ and percentage^†^ of suicides of adults aged 60+ with a current mental health problem by diagnosis and sex—National Violent Death Reporting System, 17 states, ^§^ 2007–2009.

	**Males**		**Females**		**Total**		***p* value**
**Mental health problem**	**No.**	**%**	**No.**	**%**	**No.**	**%**	
Depression/Dysthymia	1,204	80.8	444	79.1	1,648	80.4	0.351
Bipolar Disorder	93	6.2	62	11.1	155	7.6	< 0.001
Anxiety Disorder	140	9.4	67	11.9	207	10.1	0.091
Schizophrenia	23	1.5	15	2.7	38	1.9	0.091
PTSD¶	40	2.7	4	0.7	44	2.1	0.006
OCD^◊◊^	3	0.2	4	0.7	6	0.3	0.077
Eating Disorder	2	0.1	1	0.2	3	0.1	0.816
Other	89	6.0	34	6.1	123	6.0	0.849

∞ N = 2,051 (1,490 males and 561 females).

† Percentages might exceed 100% because multiple diagnosis categories might have been coded.

§ Alaska, Colorado, Georgia, Kentucky, Maryland, Massachusetts, North Carolina, New Jersey, New Mexico, Oklahoma, Oregon, Rhode Island, South Carolina, Utah, Virginia, Wisconsin and four California counties.

Posttraumatic stress disorder.

◊◊ Obsessive-compulsive disorder.

## References

[1] Centers for Disease Control and Prevention (CDC) (2009). Web-based Injury Statistics Query and Reporting System (WISQARS™).

[2] Conwell Y, Duberstein PR (2001). Suicide in elders. Ann. New York Acad. Sci.

[3] Canetto SS (1992). Gender and suicide in the elderly. Suicide Life Threaten. Behav.

[4] McIntosh JL (1992). Epidemiology of suicide in the elderly. Suicide Life Threaten. Behav.

[5] Conwell Y, Rotenberg M, Caine ED (1990). Completed suicide at age 50 and over. J. Am. Geriatr. Soc.

[6] Harwood DM, Hawton K, Hope T, Jacoby R (2001). Psychiatric disorder and personality factors associated with suicide in older people: a descriptive and case-control study. Int. J. Geriatr. Psychiatr.

[7] Harwood DM, Hawton K, Hope T, Harriss L, Jacoby R (2006). Life problems and physical illness as risk factors for suicide in older people: A descriptive and case-control study. Psychol. Med.

[8] Turvey CL, Conwell Y, Jones MP, Phillips C, Simonsick E, Pearson L, Wallace R (2002). Risk factors for late-life suicide. Am. J. Geriatr. Psychiatr.

[9] Conwell Y, Duberstein PR, Caine ED (2002). Risk factors for suicide in later life. Soc. Biol. Psychiatr.

[10] Hawton K, Appleby L, Platt S, Foster T, Cooper J, Malmberg A, Simkin S (1998). The psychological autopsy approach to studying suicide: A review of methodological issues. J. Affect. Disord.

[11] Heikkinen M, Aro H, Lonnqvist J (1994). Recent life events, social support and suicide. Acta Psychiatr Scand.

[12] Heisel MJ (2006). Suicide and its prevention among older adults. Can. J. Psychiatr.

[13] Pearson JL (1999). Studies of suicide in later life. Am. J. Geriatr. Psychiatr.

[14] Pearson JL (2006). Progress in identifying risk and protective factors in older suicidal adults. Am. J. Geriatr. Psychiatr.

[15] Horan JM, Mallonee S (2003). Injury surveillance. Epidemiol. Rev.

[16] Blazer DG, Bachar JR, Manton KG (1986). Suicide in late life. J. Am. Geriatr. Soc.

[17] Carney SS, Rich CL, Burke PA, Fowler RC (1994). Suicide over 60: The San Diego study. J. Am. Geriatr. Soc.

[18] Conner KR, Duberstein PR, Conwell Y (1999). Age-related patterns of factors associated with completed suicide in men with alcohol dependence. Am. J. Addict.

[19] Rich CL, Warsradt GM, Nemiroff RA, Fowler RC, Young D (1991). Suicide, stressors, and the life cycle. Am. J. Psychiatr.

[20] Hawton K, Harriss L (2006). Deliberate self-harm in people aged 60 years and over: Characteristics and outcome of a 20-year cohort. Int. J. Geriatr. Psychiatr.

[21] Skogman K, Alsen M, Ojehagen A (2004). Sex differences in risk factors for suicide after attempted suicide. A follow-up study of 1,052 suicide attempters. Soc. Psychiatr. Psychiatr. Epidemiol.

[22] Harwood DM, Hawton K, Hope T, Jacoby R (2000). Suicide in older people: Mode of death, demographic factors, and medical contact before death. Int. J. Geriatr. Psychiatr.

[23] Heikkinen M, Lonnqvist J (1995). Recent life events in elderly suicide a nationwide study in Finland. Int. Psychogeriatr.

[24] Centers for Disease Control and Prevention (2001). Update guidelines for evaluating public health surveillance systems: Recommendations from the guidelines working group. MMWR.

[25] Espitia-Hardeman V, Paulozzi L (2005). Injury Surveillance Training Manual.

[26] Centers for Disease Control and Prevention (CDC) (2009). National Standardized Death Certificate.

[27] Conwell Y, Thompson C (2008). Suicidal behavior in elders. Psychiatr. Clin. North Am.

[28] World Health Organization (WHO) (2007). International Statistical Classification of Diseases and Related Health Problems Tenth Revision.

[29] Luoma JB, Pearson JL (2002). Suicide and marital status in the United States, 1991–1996: Is widowhood a risk factor?. Am. J. Publ. Health.

[30] Paulozzi LJ, Mercy J, Frazier L, Annest JL (2004). CDC’s National Violent Death Reporting System: Background and methodology. Inj. Prev.

[31] Steencamp M, Frazier L, Lipskiy N, DeBerry M, Thomas S, Barker L, Karch D (2006). The National Violent Death Reporting System: An exciting new tool for public health surveillance. Inj Prev.

[32] Azreal D, Barber C, Mercy J (2001). Linking data to save lives: Recent progress in establishing a National Violent Death Reporting System. Harvard Health Pol. Rev.

[33] Centers for Disease Control and Prevention (CDC) (2009). National Violent Death Reporting System (NVDRS) Coding Manual 2009.

[34] Haw C, Hawton K (2008). Life problems and deliberate self-harm: Associations with gender, age, suicidal intent and psychiatric and personality disorder. J. Affect. Disord.

[35] Conwell Y, Duberstein PR, Connor K, Eberly S, Cox C, Caine E (2002). Access to firearms and risk for suicide in middle-aged and older adults. Am. J. Geriatr. Psychiatr.

[36] Li G (1995). The interaction effect of bereavement and sex on the risk of suicide in the elderly: An historical cohort study. Soc. Sci. Med.

[37] Younger SC, Clark DC, Oehmig-Lindroth R, Stein RJ (1990). Availability of knowledgeable informants for a psychological autopsy study of suicides committed by elderly people. J. Am. Geriatr. Soc.

[38] Centers for Disease Control and Prevention (1996). Suicide among older persons—United States, 1980–1992. MMWR.

[39] Meehan PJ, Saltzman LE, Sattin RW (1991). Suicides among older United States residents: Epidemiologic characteristics and trends. Am. J. Publ. Health.

[40] Pearson JL (1995). Suicide in late life: Challenges and opportunities for research. Int. Psychogeriatr.

[41] Conwell Y, Duberstein PR, Cox C, Herrmann J, Forbes N, Caine E (1998). Age differences in behaviors leading to completed suicide. Am. J. Geriatr. Psychiatr.

[42] McIntosh JL (1994). Generational analyses of suicide: Baby boomers and 13ers. Suicide Life Threaten. Behav.

[43] Benson RA, Brodie DC (1975). Suicide by overdoses of medicines among the aged. J. Am. Geriatr. Soc.

[44] Kirsling RA (1986). Review of suicide among elderly persons. Psychol. Rep.

[45] Victoroff VM (1984). Depression in the elderly. Ohio State Med. J.

[46] Bennewith O, Hawton K, Simkin S, Sutton L, Kapur N, Turnbull P, Gunnell D (2005). The usefulness of coroner’s data on suicides for providing information relevant to prevention. Suicide Life Threaten. Behav.

[47] US Public Health Service (2001). National Strategy for Suicide Prevention: Goals and Objectives for Action.

[48] McIntosh JL (1995). Suicide prevention in the elderly (age 65–99). Suicide Life Threaten. Behav.

